# Ptosis congénital: expérience d'un centre de soins tertiaires Marocain et mise au point

**DOI:** 10.11604/pamj.2014.19.150.3072

**Published:** 2014-10-15

**Authors:** Hanan Handor, Zouheir Hafidi, Moulayzahid Bencherif, Youssef Amrani, Adil Belmokhtar, Mina Laghmari, Rajae Daoudi

**Affiliations:** 1 Université Mohammed V Souissi, Service d'Ophtalmologie A de l'Hôpital des Spécialités, Centre Hospitalier Universitaire, Rabat, Maroc

**Keywords:** Ptosis congénital, amblyopie, désordres oculomoteurs, techniques chirurgicales, résultats esthétiques et fonctionnels, Congenital ptosis, amblyopia, oculomotor disorders, surgical techniques, aesthetic and functional results

## Abstract

Le ptosis congénital constitue la malposition palpébrale la plus fréquente de l'enfant. Le but de ce travail est de rapporter l'expérience de notre service dans la prise en charge de cette affection. Cette étude analyse les données épidémiologiques, cliniques et thérapeutiques des patients opérés pour ptosis congénital dans notre service entre Janvier 2005 et Décembre 2012. 44 patients (48 yeux) ont été opérés. La médiane d'âge était de 10 ans et une prédominance masculine a été retrouvée. Le ptosis était unilatéral et isolé dans 40 cas (90,90 %), bilatéral et associé à un syndrome de blépharophimosis dans 3 cas et à un syndrome de fibrose congénitale des muscles oculomoteurs dans un cas. Une amblyopie a été notée dans 11 cas (25%). La résection du muscle releveur de la paupière supérieure était la technique chirurgicale la plus utilisée (81,81%). Les résultats postopératoires étaient bons dans 33 cas (75%).

## Introduction

Le ptosis congénital se définit par la chute de la paupière supérieure par impotence de son muscle releveur présente dès la naissance [1]. Il s'agit de la malposition palpébrale la plus fréquente de l'enfant [2] et se présente sous des formes diverses. Sa prise en charge chirurgicale constitue une urgence lorsque la paupière supérieure barre l'axe visuel avec menace d'amblyopie [3, 4]. Plusieurs techniques chirurgicales peuvent être proposées en fonction du degré du ptosis et de la fonction du muscle releveur de la paupière supérieure. Le résultat opératoire est le plus souvent satisfaisant, cependant un certain nombre de complications postopératoires peuvent survenir. Le but de ce travail est de rapporter l'expérience de notre service dans la prise en charge du ptosis congénital. Nos résultats épidémiologiques, cliniques et thérapeutiques ont été étudiés et comparés aux données de la littérature.

## Méthodes

Il s'agit d'une étude rétrospective portant sur tous les cas de ptosis congénital pris en charge dans notre service entre Janvier 2005 et Décembre 2012. Tous les patients ont été opérés par le même chirurgien et ont eu un suivi postopératoire d'au moins 4 mois. Les patients ont bénéficié d'un examen clinique précisant le degré de la ptose de la paupière supérieure, la fonction de son muscle releveur et l'existence d'anomalies oculaires ou de syncinésies associées. La recherche d'une amblyopie en préopératoire était systématique. Le traitement chirurgical a été réalisé sous anesthésie générale et le choix de la technique chirurgicale s'est basé sur le degré de la ptose de la paupière supérieure et la fonction de son muscle releveur. La chirurgie du deuxième œil chez les malades présentant un ptosis bilatéral a été réalisée 1 mois après la chirurgie du premier œil. Nous avons réalisé un contrôle postopératoire à j1, j7, j30 puis tous les mois.

## Résultats

**Figure 1:**
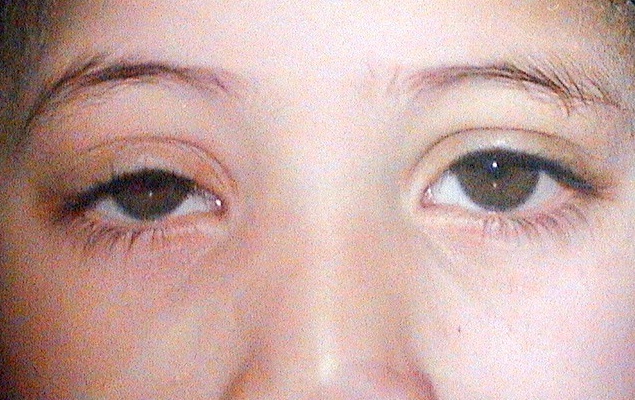
Photographie illustrant un ptosis unilatéral modéré droit chez un de nos patients

44 enfants (48 yeux) ont été opérés pour un ptosis congénital entre Janvier 2005 et Décembre 2012. La médiane d'âge dans notre série était de 10 ans, avec des extrêmes allant de 2 ans à 18 ans. Une prédominance masculine a été notée, avec un sexe ratio (H/F) de 1,75 soit 28 garçons (63,63%) opérés, pour 16 filles (36,36%). La notion de consanguinité a été retrouvée chez 5 enfants (11,36%) mais aucun antécédent familial de ptosis congénital n'a été noté chez nos malades. Le motif de consultation le plus fréquent était la gêne esthétique rapportée par les parents chez 29 patients soit 65.9 % des cas. Les 15 autres patients (34%) ont été adressés par leurs pédiatres pour avis et prise en charge. Le ptosis était unilatéral chez 40 patients (90,9%) et bilatéral chez 4 patients (9,09 %). Il était isolé dans 40 cas (90,9%), associé à un syndrome de blépharophimosis (BPES) dans 3 cas (6,81%) et à un syndrome de fibrose congénitale des muscles oculomoteurs (CFEOM) dans 1 cas (2,27%). Une amblyopie a été retrouvée chez 11 patients (25%) présentant un ptosis congénital unilatéral sévère dans 4 cas, un syndrome de CFEOM dans un cas, un syndrome de BPES dans 3 cas, une anisométropie cylindrique dans 2 cas de ptosis unilatéral modéré et une esotropie dans un cas ptosis modéré (Tableau 1). Dans notre série le degré du ptosis était minime dans 5 cas (11,36%), modéré dans 31 cas (70,45%) (Figure 1) et sévère dans 8 cas (18,18%). La fonction du RPS était nulle dans 4 cas (9,09%), faible dans 4 cas (9,09%), moyenne dans 31 cas (70,45%) et bonne dans 5 cas (11,36%).

**Tableau 1:** Récapitulatif des données épidémiologiques et cliniques de notre série

**Nombre total**	44 cas et 48 yeux
**Age**	Médiane 10 ans
**sexe**	Masculin 28 patients
	Féminin 16 patientes
**Motif de consultation**	Gêne esthétique 29 cas
	Adressé par le pédiatre 15 cas
**latéralité**	Unilatéral 40 cas
	Bilatéral 4 cas
**associations**	Isolé 40 cas
	Associés 4 cas : 3 BPES. 1 CFEOM. 11 amblyopie

**Figure 2:**
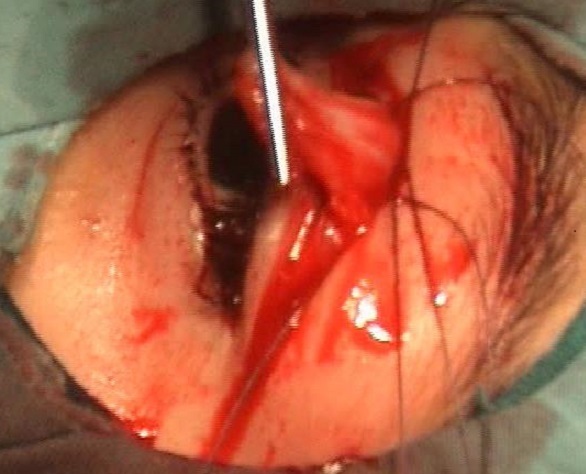
Vue peropératoire d'une résection du muscle releveur de la paupière supérieure

**Figure 3:**
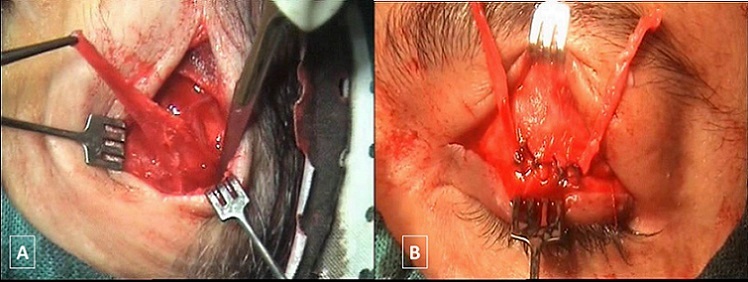
(A) prélèvement de l'aponévrose du muscle temporal. (B) vue peropératoire des 2 bandelettes de l'aponévrose du muscle temporal suturée au septum orbitaire

**Figure 4:**
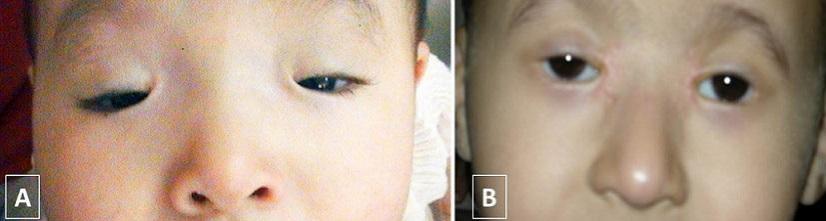
(A) aspect préopératoire d'un nourrisson avec un ptosis dans le cadre d'un syndrome de blépharophimosis. (B) photographie illustrant le résultat postopératoire chez le même malade

Les techniques chirurgicales utilisées étaient la résection du releveur de la paupière supérieure chez 36 patients (81,81 %) (Figure 2) et la suspension de la paupière au muscle frontal dans 8 cas (18,18%) (Figure 3). La suspension frontale était bilatérale dans les 3 cas de BPES (Figure 4) et dans le cas de CFEOM et unilatérale dans 4 cas de ptosis unilatéral sévère avec une fonction faible du muscle releveur (Tableau 2). Les résultats esthétiques et fonctionnels jugés sur Le degré d'ouverture de la fente palpébrale, la bonne courbure du bord libre de la paupière supérieure, la présence d'un pli palpébral bien marqué et la bonne occlusion palpébrale étaient bons dans 33 cas (75%), moyens dans 10 cas (22,72%) et médiocres dans le cas de CFEOM. Une sous correction a été obtenue chez trois patients avec nécessité d'une reprise chirurgicale. Les complications postopératoires étaient un granulome inflammatoire dans 3 cas et une cicatrice chéloïde dans 2 cas (Tableau 3). Les malades présentant une amblyopie ont été adressés en consultation de strabologie pour prise en charge.

**Tableau 2:** Répartition des malades en fonction du degré de ptosis, de la fonction du releveur de la paupière supérieure et de la technique chirurgicale utilisée

Degré du ptosis				Technique	
Mineur	5 cas	Bonne	5 cas	Résection du muscle releveur	36 cas
Modérée	31 cas	Moyenne	31 cas		
Sévère	8 cas	Faible	4 cas	Suspension au muscle frontal	8 cas
		Nulle	4 cas		

**Tableau 3:** Résultats et complications postopératoires notées dans notre série

Résultats postopératoires		Complications postopératoires
Bons	33 cas (75%)	3 sous corrections
Moyens	10 cas (22,72%)	3 granulomes inflammatoires
médiocres	1 cas (2,27%)	2 cicatrices chéloïdes

## Discussion

Le ptosis congénital constitue la malposition palpébrale la plus fréquente de l'enfant [2]. Il est souvent d'origine myogène [5, 6]. Il peut être sporadique ou familial avec généralement une transmission autosomique dominante [7]. Aucun cas de ptosis congénital familial n'a cependant été retrouvé chez nos malades. Le motif de consultation le plus fréquent dans notre série est la gêne esthétique. En effet, ce motif a été rapporté par les parents de 29 de nos patients soit 65.9 % des cas. Seuls 15 enfants ont été adressés par leur pédiatre. Ceci souligne la nécessité d'une sensibilisation du personnel médical et paramédical à cette pathologie fréquente de l'enfant.

L'âge moyen au moment du diagnostic varie en fonction des séries. Il est de 10 ans chez nos patients et est nettement plus élevé par rapport aux séries britanniques et américaines [8–10] mais proche de celui des séries maghrébines [11, 12] (Tableau 4). Ceci s'explique par le faible niveau d'instruction des parents, l'éloignement des centres médicalisés, et le manque de moyens dans nos pays. La prédominance masculine du ptosis congénital est rapportée dans plusieurs publications [9–11, 13, 14]. Dans notre série le sex ratio H/F était de 1.75. 90,90 % de nos malades avaient un ptosis isolé. Ce caractère est souvent retrouvé dans la littérature [15, 16] mais des associations du ptosis congénital à des paralysies congénitales de la IIIème paire crânienne, à la paralysie du droit supérieur [17], aux syndromes de CFEOM, de Marcus Gunn [18], et de BPES [3, 8] sont décrites. Dans notre série le syndrome de BPES a été retrouvé dans 3 cas et le syndrome de CFEOM dans un seul cas. Le ptosis congénital est une affection généralement unilatérale [1, 7, 8, 10, 12, 14, 15], c'est le cas de 90,90 % de nos malades. Nos cas de ptosis bilatéral sont représentés par les ptosis associés aux syndromes de BPES et de CFEOM.

**Tableau 4:** Tableau comparant les médianes d’âge au moment du diagnostic du ptosis congénital entre différentes séries

Série	Médiane d’âge	Nombre de patients	Pays
Lee V. et al [8]	5,5 ans	340	Royaume-Uni
Berry-Brincat et al [9]	3,88 ans	155	Royaume-Uni
Gregory et al [10]	1,3 an	107	Etats unis
Ballyout et al [11]	12 ans	11	Marrakech-Maroc
Ben Zina et al [12]	13 ans	29	Tunisie
Notre série	10 ans	44	Rabat-Maroc

L'examen clinique est une étape primordiale dans la prise en charge du ptosis congénital et nécessite un certain degré de coopération de l'enfant obtenu vers l'âge de 4 ans. Cet examen doit apprécier le degré du ptosis et la fonction du muscle releveur de la paupière supérieure. Les formes modérées de la ptose avec une fonction moyenne du releveur étaient prédominantes dans notre série. Une étude algérienne souligne également la prédominance de ces formes moyennes par rapport aux formes mineures et majeures [19]. L'examen clinique doit aussi rechercher une amblyopie. Celle-ci est retrouvée en cas d'anisométropie, de strabisme [20], d'un fort astigmatisme [21] ou en présence d'un ptosis majeur obturant l'axe visuel [22]. Nous avons retrouvé dans notre série 11 cas d'amblyopie (25%) en rapport avec un strabisme dans un cas de ptosis unilatéral modéré, avec une anisométropie cylindrique dans 2 cas de ptosis unilatéral modéré et dans le cadre des syndromes de BPES et de CFEOM. Une amblyopie de privation a été notée dans 4 cas de ptosis majeur unilatéral. L'importance de l'examen clinique réside également dans l'évaluation du degré de tolérance à l'acte chirurgical. Il faut en effet rechercher l'absence d'occlusion palpébrale par une brièveté de la paupière supérieure et l'absence du signe de Charles Bell puisqu'ils constituent des facteurs de risque d'exposition cornéenne postopératoire [15] donnant lieu à de nombreuses complications pouvant compromettre la fonction visuelle.

Le traitement du ptosis congénital est exclusivement chirurgical [2]. Trois questions sont importantes à considérer lors de la prise en charge d'un ptosis congénial à savoir: Qui opérer? Quand opérer? Et quelle technique utiliser? En effet, les indications chirurgicales peuvent être, d'une part fonctionnelles devant le risque d'altération du développement de la fonction visuelle quand le ptosis barre l'axe visuel, et d'autre part cosmétiques [8].

Les enfants présentant un ptosis congénital sont souvent opérés à l'âge de 3 ans ou 4 ans avant l'entrée à l'école [3, 23] cependant; Lorsque le ptosis est amblyogène du fait de son unilatéralité et de son importance, l'intervention constitue une urgence et est réalisée au cours des premiers mois de vie [21]. Dans les cas associés à des anomalies oculomotrices (strabisme, une paralysie de la verticalité, une paralysie oculomotrice) la chirurgie du ptosis n'est proposée qu'après avoir obtenu un recentrage du globe en position primaire [3, 23] sauf en cas de risque important d'amblyopie. Les cas de BPES, s'opèrent en 2 temps. La correction du télécanthus précède la chirurgie du ptosis sauf en cas de risque majeur d'amblyopie [24]. Dans notre série l'indication d'une correction urgente du ptosis vu le risque d'amblyopie a été posée dans 7 cas.

La chirurgie du ptosis chez l'enfant se fait sous anesthésie générale contrairement à l'adulte, rendant l'appréciation per opératoire du degré d'ouverture de la fente palpébrale plus difficile à réaliser. Le choix de la technique chirurgicale se base sur les données de l'examen clinique (degré du ptosis et fonction du muscle releveur). Il est préférable, chaque fois que possible, de réaliser une chirurgie du releveur et de réserver les techniques de suspension aux releveurs inexploitables. L'analyse des résultats de notre série nous permet de noter que la résection du muscle releveur de la paupière supérieure a été la technique chirurgicale la plus utilisée (81,81% des cas), suivie de la suspension au muscle frontal (18,18 %).

Cette dernière technique fait appel à différents matériaux de suspension. Ces matériaux peuvent être synthétiques (polytétrafluoroéthylène ou PTFE, fil de silicone, nylon, polyester, propylène [25, 26] ou autogènes (le fascia lata et l'aponévrose du muscle temporal) [15]. Plusieurs travaux ont comparé les résultats de la suspension frontale en utilisant les différents matériaux précédemment cités. Certains auteurs préfèrent utiliser les greffes autogènes et en particulier les greffes de fascia lata; car elles assurent des résultats meilleurs, plus durables dans le temps, avec très peu de risques d'infection [26–28]. Pour d'autres, l'utilisation de greffes autogènes ou synthétiques aboutit à des résultats esthétiques et fonctionnels comparables [29]. Selon KENGO HAYASHI et al l'utilisation du PTFE expose à un faible taux de récidives et de complications [30]. Dans notre série les 3 cas de granulomes inflammatoires postopératoires ont été notés chez des malades ayant bénéficié d'une suspension avec du matériel synthétique. Nous avons utilisé dans les autres cas de suspension l'aponévrose du muscle temporal que nous jugeons accessible et très bien tolérée. Les résultats postopératoires dans notre série, étaient bons dans 33 cas (75%), moyens dans 10 cas (22,72%) % et médiocre avec persistance du ptosis dans le cas de CFEOM. Trois cas de sous corrections ont été notés chez nos malades avec la nécessité d'une reprise chirurgicale. L'exposition cornéenne postopératoire n'est survenue chez aucun malade du fait d'une chirurgie réglée évitant les sur-corrections et grâce à la mise en place systématique d'un fil de traction au niveau de la paupière inférieure en fin d'intervention assurant l'occlusion palpébrale dans les 24 heures suivant la chirurgie.

## Conclusion

La prise en charge du ptosis congénital doit se faire dans des centres spécialisés en chirurgie orbito palpébrales et dotés d'une équipe d'ophtalmo pédiatrie afin d'optimiser les résultats thérapeutiques. Sa prise en charge s'appuie sur certains points essentiels: un bon examen clinique préopératoire jugeant l'urgence de la chirurgie et guidant le choix de la technique chirurgicale, une chirurgie réalisée par un chirurgien expérimenté, un suivi postopératoire régulier guettant et gérant les complications.
